# Role of Reuniens Nucleus Projections to the Medial Prefrontal Cortex and to the Hippocampal Pyramidal CA1 Area in Associative Learning

**DOI:** 10.1371/journal.pone.0023538

**Published:** 2011-08-15

**Authors:** Lyndell Eleore, Juan Carlos López-Ramos, Rafael Guerra-Narbona, José M. Delgado-García

**Affiliations:** División de Neurociencias, Universidad Pablo de Olavide, Sevilla, Spain; Pontifical Catholic University of Rio Grande, Brazil

## Abstract

We studied the interactions between short- and long-term plastic changes taking place during the acquisition of a classical eyeblink conditioning and following high-frequency stimulation (HFS) of the reuniens nucleus in behaving mice. Synaptic changes in strength were studied at the reuniens-medial prefrontal cortex (mPFC) and the reuniens-CA1 synapses. Input/output curves and a paired-pulse study enabled determining the functional capabilities of the two synapses and the optimal intensities to be applied at the reuniens nucleus during classical eyeblink conditioning and for HFS applied to the reuniens nucleus. Animals were conditioned using a trace paradigm, with a tone as conditioned stimulus (CS) and an electric shock to the trigeminal nerve as unconditioned stimulus (US). A single pulse was presented to the reuniens nucleus to evoke field EPSPs (fEPSPs) in mPFC and CA1 areas during the CS-US interval. No significant changes in synaptic strength were observed at the reuniens-mPFC and reuniens-CA1 synapses during the acquisition of eyelid conditioned responses (CRs). Two successive HFS sessions carried out during the first two conditioning days decreased the percentage of CRs, without evoking any long-term potentiation (LTP) at the recording sites. HFS of the reuniens nucleus also prevented the proper acquisition of an object discrimination task. A subsequent study revealed that HFS of the reuniens nucleus evoked a significant decrease of paired-pulse facilitation. In conclusion, reuniens nucleus projections to prefrontal and hippocampal circuits seem to participate in the acquisition of associative learning through a mechanism that does not required the development of LTP.

## Introduction

Classical eyeblink conditioning, using a trace paradigm, is an experimental model commonly used to evaluate hippocampal involvement in associative learning processes [1], [2]. Hippocampal removal or malfunctioning severely impairs the acquisition of CRs across training [3], [4]. Moreover, the LTP induced by HFS of Schaeffer collaterals also blocks the acquisition process [2], [5]. In humans, it has been shown that trace conditioning requires a declarative (explicit) memory [6] and/or conscious knowledge [7] of established relationships between CS and US.

The mPFC is functionally connected with the hippocampus. In fact, the hippocampus (CA1 ventral and subiculum) projects strongly to the ventral mPFC [8], [9]. Pyramidal CA1 neurons contact monosynaptically and form asymmetrical synapses with mPFC pyramidal cells and interneurons [10]. In addition, electrical stimulation of the CA1 layer evokes EPSPs in the mPFC, and high/low frequency stimulations of the hippocampal field produce LTP/long-term depression (LTD) respectively in the mPFC [11]–[15]. Finally, it has been demonstrated that electrical stimulation of the mPFC also prevents the expression of conditioned eyeblinks in rabbits [16].

Apart from a few projections from the mPFC to parahippocampal structures (including the entorhinal cortex), there is no direct projection from the mPFC to the hippocampus. This raises the question of how the mPFC can influence hippocampal activity. In this regard, the reuniens nucleus represents an important source of thalamic input to the hippocampus and to the entorhinal cortex, and is strongly interconnected with the mPFC [17]–[20].

Here, we have studied reuniens nucleus contribution to associative learning, using the classical eyeblink conditioning with a trace paradigm. For this, a tone as a CS and an electric shock to the supraorbital nerve as a US were presented to the animals. CRs were quantified from the electromyographic (EMG) activity of the orbicularis oculi muscle. We also recorded the field excitatory postsynaptic potential (fEPSP) evoked in hippocampal CA1 pyramidal cells and in the prelimbic area of the mPFC by single pulses presented to the reuniens nucleus within the CS–US interval. An object recognition task was also used as it is assumed that the prefrontal cortex is critical in determining the spatial organization of the brain's object representations. Finally, in an attempt to determine the impact of synaptic plasticity of the reuniens nucleus in the acquisition process, high-frequency stimulation (HFS) was applied to selected groups during the classical conditioning experiment to determine whether these pathways were capable of evoking an LTP of the involved synapses and/or of preventing the learning process. Results indicate that the reuniens nucleus participates in the acquisition of classical eyeblink conditioning presumably through presynaptic mechanisms involving its projections to the mPFC and to the pyramidal CA1 area.

## Results

### EMG and synaptic field potentials recorded in alert behaving mice

**Figure 1:**
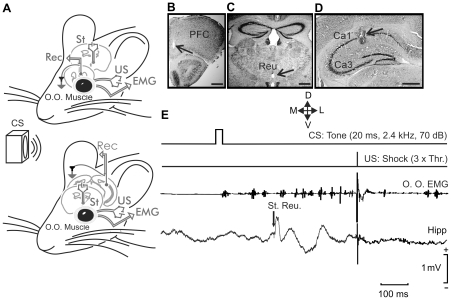
Experimental design. (A) EMG recording electrodes were implanted in the orbicularis oculi (O.O.) muscle of the upper left eyelid. In addition, bipolar stimulating electrodes were implanted on the ipsilateral supraorbital nerve for presentation of unconditioned stimulus (US). The conditioned stimulus (CS) consisted of a tone delivered from a loudspeaker located 30 cm from the animal's head. Animals were also implanted with stimulating electrodes in the thalamic reuniens nucleus and with recording electrodes in the medial prefrontal cortex (top diagram) or the hippocampal CA1 area (bottom diagram). (B–D) Photomicrographs illustrating the location of recording electrodes in the mPFC (B) and in the hippocampal CA1 area (D), as well as the stimulation (C) site (arrows). Calibration bars 500 µm. Abbreviations: D, L, M, V, dorsal, lateral, medial, and ventral. E, A schematic representation of the conditioning paradigm, illustrating CS and US stimuli, and the moment at which a single pulse (100 µs, square, biphasic) was presented to the reuniens nucleus (St. Reu.). An example of an EMG record from the orbicularis oculi (O.O.) muscle obtained from the 9th conditioning session is illustrated, as well as an extracellular record of hippocampal activity from the same animal, session, and trial. Note the fEPSPs evoked by the pulse presented to the reuniens nucleus.

Following previous descriptions [2], [5] and as illustrated in Figure 1, the experimental design used in this study enables the simultaneous recording of classically conditioned eyelid responses and of fEPSPs evoked at selected thalamo-cortical (reuniens-CA1 and reuniens-mPFC) synapses (Fig. 1*A*–*D*). Stimulating and recording electrodes implanted in the upper lid did not seem to disturb its normal kinematics, and allowed the generation of reflex and conditioned eyeblinks (Fig. 1*E*). Conditioned eyeblinks were easily distinguished in EMG recordings, and were quantified following criteria described previously [2], [21]. The evolution of reuniens-CA1 and reuniens-mPFC field synaptic potentials in simultaneity with the acquisition of CRs was recorded. The electrical stimulation of the ipsilateral reuniens nucleus presented 250 ms after the CS evoked a definite fEPSP in the pyramidal CA1 area (Fig. 1*E*) or in the mPFC. Field EPSPs recorded with this procedure do not present any spontaneous tendency toward a sustained increase or decrease [5], [22]. Only data collected from electrodes located at the selected (CA1 and mPFC) sites (Fig. 1*B*–*D*) were further analyzed and included in this study.

### Input/output curves and paired-pulse facilitation characteristic of reuniens-mPFC and reuniens-CA1 synapses

**Figure 2:**
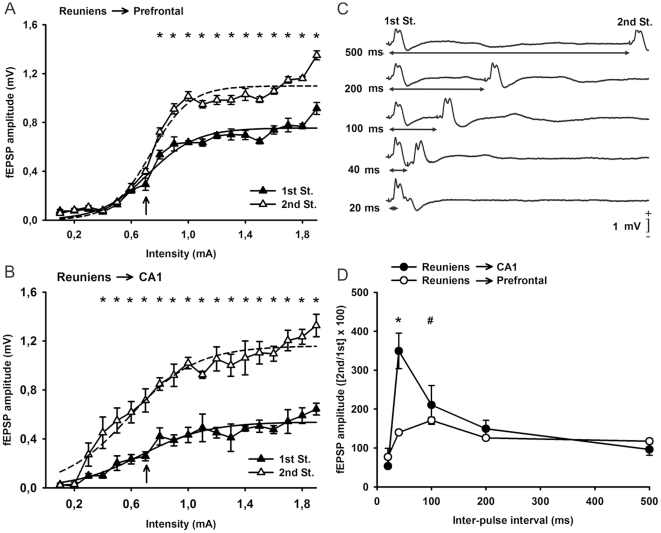
Input/output curves and paired-pulse stimulation of the reuniens-mPFC and reuniens-CA1 synapses using paired-pulse stimulation. (A) Relationships between the intensity (in mA) of pairs of stimuli (40 ms of interstimulus interval) presented to the reuniens nucleus and the amplitude of the fEPSPs evoked in the mPFC by the 1st (black triangles) and the 2nd (white triangles) pulses. Data are represented as mean ± s.e.m. *, *P*<0.01 for differences in the amplitude of fEPSPs evoked by the two pulses. The best three-parameter sigmoidal fits to the two set of data are included (1st pulses, continuous line: y = 0.75/(1+exp[−(x-0.71)/0.16]); r = 0.97; *P*<0.001; 2nd pulses, dashed line: y = 1.09/(1+exp[−(x-0.73)/0.13]); r = 0.98; *P*<0.001). *, *P*<0.01. (B) The same as for *A*, but representing data collected from the reuniens-CA1 synapse. *P<0.01 for differences between fEPSPs evoked by the two pulses. Sigmoidal fits to the two set of data are also included (1st pulses, continuous line: y = 0.53/(1+exp[−(x-0.67)/0.23]); r = 0.96; *P*<0.001; 2nd pulses, dashed line: y = 1.16/(1+exp[−(x-0.58)/0.23]); r = 0.97; *P*<0.001). *, *P*<0.01. (C) Representative records (average of three traces) of fEPSPs evoked at the reuniens-mPFC synapse by paired-pulse stimulation at five different (20, 40, 100, 200, and 500 ms) interpulse intervals. Stimulus intensity was 0.7 mA, i.e., ≈50% of the asymptotic value for this synapse (see arrow in *A*). (D) Paired-pulse depression and facilitation of fEPSPs recorded from the reuniens-mPFC (white circles) and the reuniens-CA1 (black circles) synapses. The pairs of pulses were set at 0.7 mA (see arrows in *A* and *B*). The data shown are mean ± s.e.m. amplitudes of the 2nd fEPSP expressed as a percentage of the 1st [(2nd/1st) ×100] for the five interstimulus intervals used in this study. Note that peak facilitation was at 40 ms of interval for the reuniens-CA1 synapse and at 100 ms for the reuniens-mPFC synapse. *, statistical differences for reuniens-CA1 synaptic facilitation at 40 ms of interval, with respect to the 500 ms one; #, statistical differences for reuniens-mPFC synaptic facilitation at 100 ms interval, with respect to both the 20 and 500 ms ones, *P*<0.05. Tukey's post-hoc comparison test.

In a first series of experiments, and following previous descriptions [20], we studied the changes in the amplitude of fEPSPs evoked in the mPFC (Fig. 2*A*) and in the pyramidal CA1 area (Fig. 2*B*) by paired-pulse (40 ms interstimulus interval) stimulation of the ipsilateral reuniens nucleus at increasing intensities. As illustrated in Fig. 2*A*, the amplitude of fEPSPs (in mV) evoked in the mPFC by the 1st pulse (black triangles) increased steadily with current strength until reaching asymptotic values. In contrast, fEPSPs evoked by the 2nd pulse increased more or less in parallel with the fEPSPs evoked by the 1st pulse, but with larger values, mainly at higher stimulus intensity. Indeed, fEPSP amplitudes evoked by the 2nd pulse were significantly larger than those evoked by the 1st [F_(1,7)_ = 46,16; *P*<0,001, for asterisks illustrated in Fig. 2*A*]. Interestingly, both curves presented a sigmoid-like shape (best curve fits are indicated in the figure legend). Similar displays were obtained for data collected from the reuniens-CA1 synapse (Fig. 2*B*) — namely, the paired-pulse facilitation evoked at low stimulus intensities (<0.6 mA) was increased in response to higher stimulus intensities (>0.6 mA). Significant differences [F_(1,4)_ = 534,12; *P*<0,001, for asterisks illustrated in Fig. 2*B*] between fEPSPs evoked by the 1st and the 2nd pulse were evident for a wide range (0.6–1.8 mA) of stimulus intensities.

In addition, we studied the changes in amplitude of fEPSPs evoked in the mPFC and in the pyramidal CA1 area by paired-pulse stimulation of the ipsilateral reuniens nucleus at a fixed intensity (0.7 mA for both synapses, see arrows in Fig. 2*A, B*), but at increasing intervals (10, 20, 40, 100, 200, and 500 ms, see Fig. 2*C, D*). Maximum facilitation for the reuniens-mPFC synapse was observed at 100 ms of paired-pulse interval, whilst the maximum facilitation for the reuniens-CA1 synapse took place at 40 ms of paired-pulse interval (Fig. 2*D*).

### Effects on the acquisition of conditioned eyeblinks and on fEPSPs evoked at the reuniens-mPFC synapse following HFS of the reuniens nucleus during training

The aim of this second series of experiments was to determine the effects of HFS of the reuniens nucleus on the acquisition of conditioned eyeblink responses and on the amplitude of fEPSPs evoked at the reuniens-mPFC synapse. In this regard, it has already been reported [2], [5] that HFS of Schaffer collaterals interferes with the acquisition of a classical eyeblink conditioning at the same time that it evokes a significant and long-lasting (i.e., days) LTP in the ipsilateral pyramidal CA1 area. In those two studies, it was also shown that there is an activity-dependent increase in the synaptic strength of the CA3-CA1 synapse evoked across the successive conditioning sessions. Thus, we checked here whether similar effects could be detected at the reuniens-mPFC synapse.

**Figure 3:**
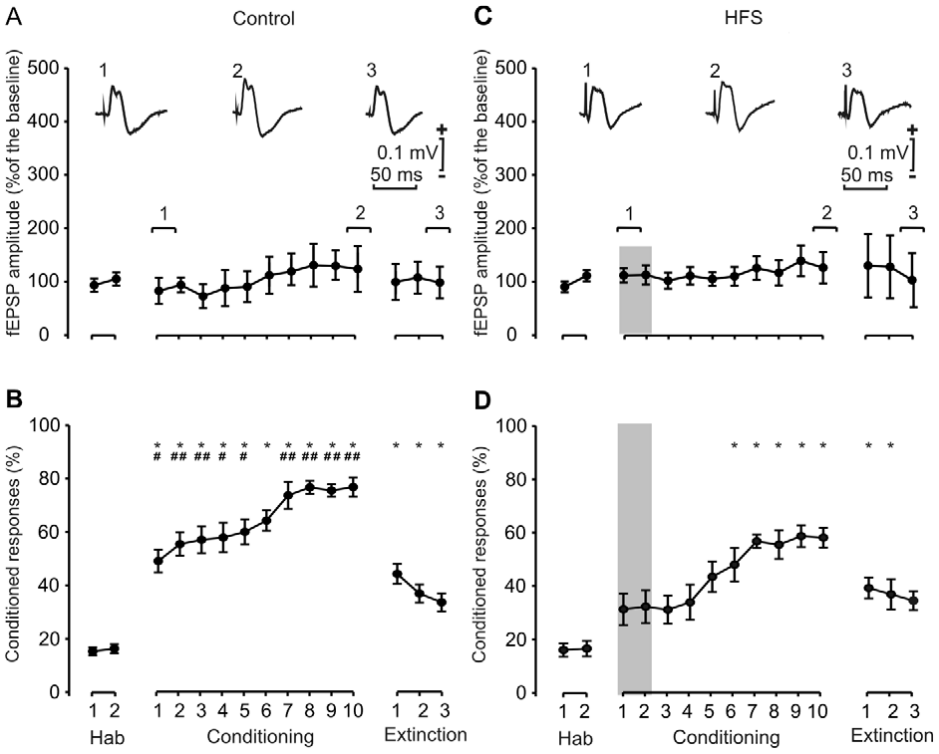
Learning curves and evolution of synaptic field potentials evoked in the PFC by electrical stimulation of the reuniens nucleus for controls (A, B) and following two HFS sessions (C, D). (A) Evolution of fEPSPs evoked at the reuniens-PFC synapse across the successive habituation, conditioning, and extinction sessions. At the top are illustrated selected fEPSPs recorded in the PFC during the indicated sessions, following a single pulse presented to the reuniens nucleus. Note that no significant change in fEPSP amplitude was observed across conditioning. Calibrations as indicated. (B) Evolution of the percentage (%) of conditioned responses during the successive sessions. Mean % values are followed by ± s.e.m. (C) Evolution of fEPSPs evoked at the reuniens-PFC synapse across training, following two HFS sessions presented 30 min before the first two conditioning sessions. Note that these HFS sessions did not evoke any noticeable LTP in fEPSPs recorded in the PFC. (D) Evolution of the percentage (%) of conditioned responses during the successive sessions following the two HFS sessions. Note the small increase in the percentage of conditioned responses. *, *P*<0.001, significant differences with respect to habituation values for B and D. #, *P*<0.05; ##, *P*<0.01, significant differences between the percentage of conditioned responses achieved without (B) or following two HFS sessions (D).

As illustrated in Figure 3*B*, classical eyeblink conditioning was easily achieved by mice. Indeed, this group of animals reached ≈60% of conditioned responses by the 5th-6th conditioning sessions, and asymptotic values were >70% of conditioned responses from the 7th session on. The percentage of conditioned responses was significantly larger [F_(10,69)_ = 33,82; *P*<0,001] than values reached during the habituation sessions from the 2nd to the 10th, although no significant activity-dependent change in synaptic strength was noticed in the amplitude of the fEPSPs evoked at the reuniens-mPFC synapse across conditioning sessions. This result suggested that the reuniens-mPFC synapse is not involved in this type of associative learning.

Moreover, when an HFS protocol was applied during the first two conditioning sessions, the percentages of conditioned eyeblink responses presented by the animals (Fig. 3*D*) were significantly [F_(1,11)_ = 10,7 ; *P*<0,01] lower than values reached by the control group (Fig. 3*B*,). Thus, percentages of ≈60% of conditioned responses were reached only during the 8th conditioning session, and asymptotic values never reached values >60%. Surprisingly, the amplitude of fEPSPs evoked in the mPFC area by single electrical stimuli presented to the reuniens nucleus was not significantly [F_(1,12)_ = 0,96; *P* = 0,34] modified by the two HFS protocols applied. Thus, the effects on associative learning of HFS protocols presented to the reuniens nucleus could not be ascribed to LTP changes evoked at the reuniens-mPFC synapse.

### Effects on the acquisition of conditioned eyeblinks and on fEPSPs evoked at the reuniens-CA1 synapse following HFS of the reuniens nucleus during training

**Figure 4:**
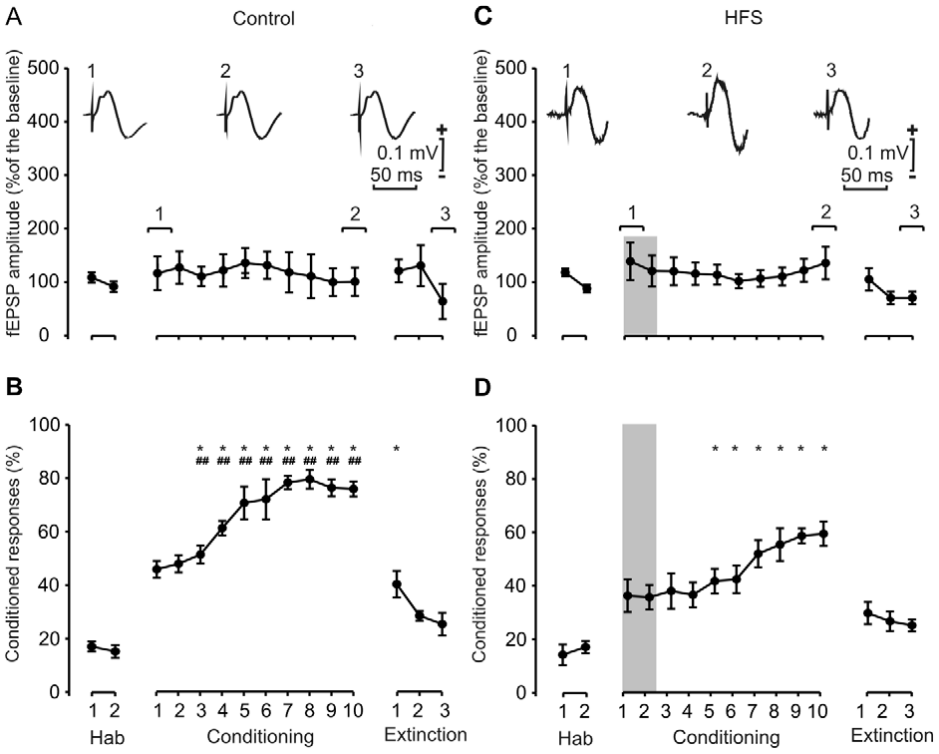
Learning curves and evolution of synaptic field potentials evoked in the CA1 area by the electrical stimulation of the reuniens nucleus for controls (*A, B*) and following two HFS sessions (C, D). *(*A) Evolution of fEPSPs evoked at the reuniens-CA1 synapse across the successive habituation, conditioning, and extinction sessions. At the top are illustrated selected fEPSPs recorded in the CA1 area during the indicated sessions, following a single pulse presented to the reuniens nucleus. Note that no significant change in fEPSP amplitude was observed across conditioning. Calibrations as indicated. (B) Evolution of the percentage (%) of conditioned responses during the successive sessions. Mean % values are followed by ± s.e.m. (C) Evolution of fEPSPs evoked at the reuniens-CA1 synapse across training, following two HFS sessions presented 30 min before the first two conditioning sessions. Note that these HFS sessions did not evoke any noticeable LTP in fEPSPs recorded in the CA1 area. (D) Evolution of the percentage (%) of conditioned responses during the successive sessions following the two HFS sessions. Note the small increase in the percentage of conditioned responses. *, *P*<0.001, significant differences with respect to habituation values for B and D. #, *P*<0.05; ##, *P*<0.01, significant differences between the percentage of conditioned responses achieved without (B) or following two HFS sessions (D).

We decided to repeat the set of experiments shown in Figure 3, but this time checking the effects evoked at the reuniens-CA1 synapse by classical eyeblink conditioning, and by presenting two HFS protocols during the first two conditioning sessions. As illustrated in Figure 4*B*, this new group of mice also reached ≈60% of conditioning responses by the 4th session, and their asymptotic values were >70% for the 7th-10th sessions. Indeed, the percentage of conditioned responses was significantly [F_(10,73)_ = 38,82; *P*<0,001] larger than values collected from the two habituation sessions from the 1st to the 10th conditioning sessions (Fig. 4*B*). However, in this case also there was no significant [F_(9,86)_ = 0,16; *P* = 0,99] change in the amplitude of fEPSPs evoked at the reuniens-CA1 synapse across training (Fig. 4*A*).

Also in this case, when an HFS protocol was applied during the first two conditioning sessions, the percentages of conditioned eyeblink responses presented by the animals (Fig. 4*D*) were significantly [F_(1,12)_ = 28,68 ; *P*<0,001] lower than values reached by the control group (Fig. 4*B*). Thus, percentages of ≈60% of conditioned responses were reached only during the 8th-10th conditioning sessions, and asymptotic values never reached values >70%. Similarly to what was found for the reuniens-mPFC synapse, the amplitude of fEPSPs evoked at the CA1 by single electrical stimuli presented to the reuniens nucleus was not significantly [F_(1,12)_ = 2,01 ; *P* = 0,182] modified by the two HFS protocols applied to the reuniens nucleus. Here again, the effects on associative learning of HFS protocols presented to the reuniens nucleus could not be ascribed to LTP changes evoked at the reuniens-CA1 synapse.

### Effects of HFS of the reuniens nucleus on an object recognition test

**Figure 5:**
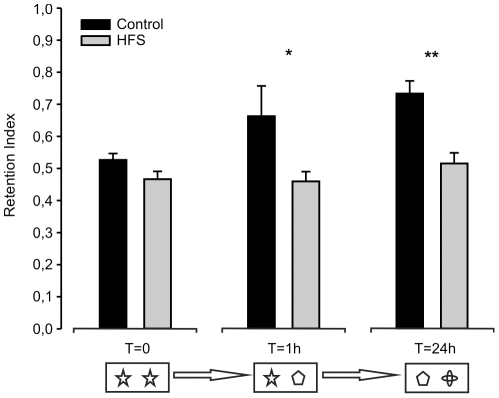
Results collected from the object recognition task. A representation of the attention devoted to a familiar object or to a novel one by control mice (black bars) and by animals following a single HFS session (gray bars), during an object recognition task, for the training (T = 0) session, and 1 h (T = 1) and 24 h (T = 24) afterwards. The object presentation sequence is schematized at the bottom. Values are mean ± s.e.m. of the percentage of the total attention exhibited in each session. *, Statistical differences between percentages of attention, *P*<0.05; **, *P*<0.01.

In another series of experiments, we determined the learning capabilities of mice in an object recognition task following a single HFS protocol presented to the reuniens nucleus. As illustrated in Figure 5, during the acquisition period the two groups of animals spent similar amounts of time (about 50%) exploring two identical objects (O1 and O2), indicating no spatial preferences associated with their location. Since the total time of approach to the two objects varied considerably in the different animals, we preferred to use percentage of attention as a quantitative index. The retention index was defined as the time spent exploring the new object divided by the time spent exploring both objects and multiplied by 100 [23]. The percentage of attention during the acquisition period was similar for both control and HFS groups [F_(1,12)_ = 3,27 ; *P* = 0,095].

During the first choice trial, 1 h after the initial training session, mice were allowed to explore a novel object (B1) and a familiar one (O3). In this case, the analysis of variance of the collected data indicated that control mice presented a significant difference in retention index, compared with the HFS group [F_(1,12)_ = 6,027 ; *P*<0,05; Fig. 5].

During the second choice trial, carried out 24 h after the acquisition period (Fig. 5, T = 24 h session), animals were presented with a novel object (C1) and a familiar one (B2). In this case again, an increase in the percentage of attention spent exploring the novel object was found for controls, while the HFS group failed to spend more time exploring the novel versus the familiar object [F_(1,12)_ = 17,93 ; *P*<0,01].

In summary, and as already described for classical eyeblink conditioning, HFS mice were incapable of performing the object recognition tasks properly, as compared with the control group. Similar deficits in the performance of an object recognition test have recently been reported following HFS of the CA3-CA1 synapse in wild-type mice [24].

### Evolution of fEPSPs evoked in the PFC (*A*) and in the CA1 area (*B*) by paired-pulse stimulation of reuniens nucleus before and after two HFS sessions

In an additional series of experiments, we checked whether HFS protocols presented at higher intensities (i.e., at 50% of the amount necessary to evoke asymptotic fEPSP responses in the input/output curves, see Fig. 2*A, B*) were capable of evoking LTP responses at the reuniens-mPFC and/or the reuniens-CA1 synapses. Moreover, it has recently been reported that the double-pulse test can be successfully used to determine presynaptic components present in LTP processes evoked at cortical synapses in behaving mice [25].

**Figure 6:**
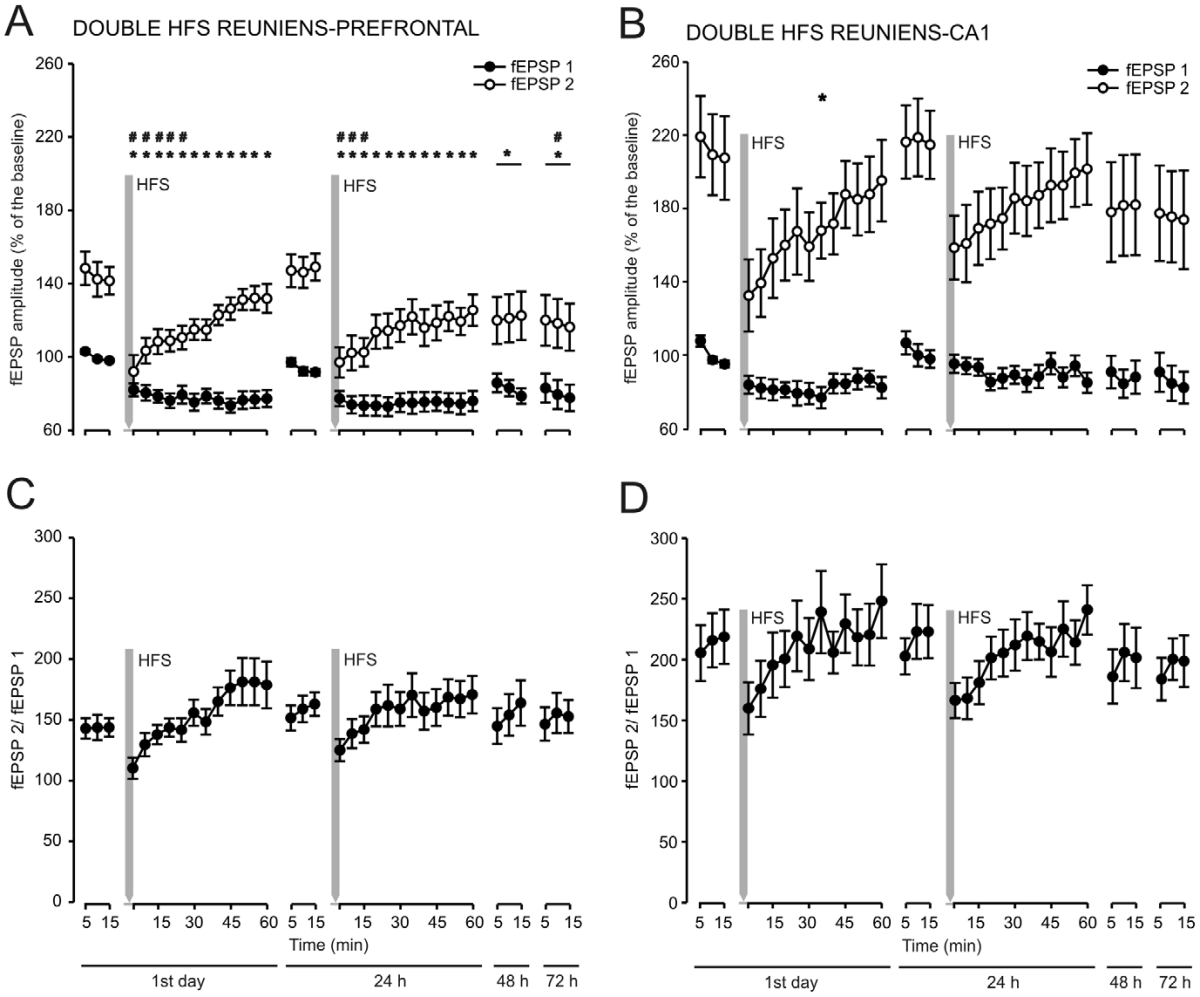
Effects of two HFS sessions on the reuniens-CA1 and reuniens-mPFC synapses. (A, B) Evolution of fEPSPs evoked in the PFC (A) and in the CA1 area (B) by paired-pulse stimulation of reuniens nucleus before and after two HFS sessions. Each animal was presented with two HFS sessions (see shaded areas) each consisting of five 200 Hz, 100 ms trains of pulses at a rate of 1/s. This protocol was presented six times, at intervals of 1 min. The 100 µs, square, biphasic pulses used to evoke LTP were applied at the same intensity used for the single pulse presented following HFS presentation. The evolution of LTP was checked using a pair of pulses (1st, black circles; 2nd, white circles) with an interstimulus interval of 40 ms. Recording was carried out for 72 h. Note that fEPSP amplitudes evoked by the 1st and the 2nd pulses reached values below baseline following the two HFS sessions for both synapses. *, *P*<0.05 for fEPSPs evoked by the 1st pulse; #, *P*<0.05 for fEPSPs evoked by the 2nd pulse.

As illustrated in Fig. 6, the two HFS protocols presented to the reuniens nucleus evoked an LTD-like phenomenon at both reuniens-mPFC (Fig. 6*A*) and reuniens-CA1 (Fig. 6*B*) synapses. However, the decrease in the amplitude of fEPSPs evoked by the 1st pulse was significantly different from values collected during baseline records particularly for the reuniens-mPFC synapse [F_(27,352)_ = 3,65 ; *P*<0,001], but also for the reuniens-CA1 one [F_(27,323)_ = 1,66; *P*<0,05].

In contrast, the two strong HFS sessions evoked significant [F_(27,352)_ = 3,36; *P*<0,001] changes in the amplitude of the fEPSP evoked by the 2nd pulse only for the reuniens-mPFC synapse. Thus, the amplitude of the fEPSP evoked by a 2nd pulse applied to the reuniens-mPFC synapse was decreased after the 1st and after the 2nd HFS sessions (Fig. 6*A*), and was recovered 72 h later. Although less evident, the same effect was also noticeable at the reuniens-CA1 synapse (Fig. 6*B*).

Obviously, these differential effects on fEPSPs evoked by the 1st and 2nd pulses produced a significant change in the relationship (2nd fEPSP/1st EPSP) ×100 (Fig. 6*A, B*). As indicated above, changes in this relationship are indicative of neural phenomena taking place at the presynaptic site [26]–[29]. In this regard, it has been shown recently that significant changes in this relationship are noticeable at the CA3-CA1 synapse across associative learning and following HFS protocols — i.e., across LTP evolution [25].

Although the slope of fEPSPs evoked by the 2nd pulse with respect to the 1st was significantly larger across the three days of recordings [F_(27,352)_ = 4,19; *P*<0,001], there were noticeable changes in the paired-pulse facilitation after the presentation of the two HFS sessions for the reuniens-mPFC synapse (Fig. 6). Giving a value of 100% to fEPSPs evoked by the 1st pulse (black circles in Fig. 6*A, B*), the paired-pulse facilitation evoked by the 2nd pulse (white circles, Fig. 6 *A, B*) during baseline records was ≈140% for the reuniens-mPFC synapse and ≈220% for the reuniens-CA1 synapse. This facilitation was reduced to 111% when measured 15 min after the first HFS session for the reuniens-mPFC synapse and to 159,3% for the reuniens-CA1 synapse. Something similar occurred following the second HFS session (Fig. 6). That is, the paired-pulse facilitation was significantly ([F_(12,142)_ = 8,48; *P*<0,001] and [F_(12,148)_ = 2,56; *P*<0,01] for the reuniens-mPFC and the reuniens-CA1 synapses, respectively) decreased for the two synapses following the 1st two HFS protocols, and also [F_(14,110)_ = 3,18; *P*<0,001] for the reuniens-mPFC synapse following the 2nd HFS protocol.

Following both HFS sessions, the paired-pulse facilitation ratio increased steadily across time with a low slope (y = 1,15+0,06x; r = 0,96; *P*<0,0001 and y = 1,71+0,05x; r = 0,83; *P* = 0,0007, for the reuniens-mPFC and the reuniens-CA1 synapses, respectively, following the 1st HFS protocol, and y = 1,35+0,03x; r = 0,84; *P* = 0,0006 and y = 1,67+0,05x; r = 0,89; *P*<0,0001, for the reuniens-mPFC and the reuniens-CA1 synapses, respectively, following the 2nd HFS protocol).

In conclusion, strong HFS protocols presented to the reuniens nucleus modify the amplitude of fEPSPs evoked by a 1st pulse at the reuniens-mPFC and reuniens CA1 synapses. Moreover, the two HFS sessions evoked significant changes in the paired-pulse facilitation characterizing the two synapses.

## Discussion

### General comments regarding the present findings

There is scarce information with regard to the role of midline thalamic nuclei in the acquisition and storage of new motor and cognitive abilities. In fact, there are important circuits interconnecting prefrontal and hippocampal neural centers across, among others, the reuniens nucleus. Thus, it should be expected that this thalamic nucleus plays a role in learning and memory processes. Here, we have studied input/output curves and paired-pulse modulation present in reuniens-prefrontal and reuniens-CA1 synapses. In short, the paired-pulse facilitation observed in these two different synapses yielded values similar to those recorded in behaving mice at the CA3-CA1 synapse, using similar stimulation and recording procedures [25]. Interestingly, HFS of the reuniens nucleus prevented the proper acquisition of classical eyeblink conditioning using a trace paradigm (a typical associative learning task involving hippocampal circuits) and of an object discrimination test (a cognitive task involving both hippocampal and prefrontal structures). In this regard, it has recently been reported that LTP evoked at the hippocampal CA3-CA1 synapse is able to interfere with the acquisition of classical eyeblink conditioning [2] and of an object recognition test [24], but in the present case, HFS of the reuniens nucleus interfered with the learning process without presenting any sign of LTP evoked at the two studied synapses (reuniens-mPFC and reuniens-CA1).

The observation that HFS applied to the reuniens nucleus interferes the acquisition of conditioned eyelid responses is a new finding, and suggest that this nucleus is involved in learning and memory processes, by a still unknown mechanism. In accordance with previous studies carried out on reuniens-CA1 and reuniens-mPFC synapses [20], [30], we decided to take advantage here of paired pulse stimulation of the reuniens nucleus. Paired-pulse stimulation is a form of short-term synaptic modulation frequently used as an indirect measurement of changes in the probability of release of neurotransmitter at the presynaptic terminal [28]. Hence, any change in the response evoked by the 2nd stimulus in relation to the 1st will be indicative of a presynaptic action [27]. Thus, these data suggest that the deleterious effects on associative and cognitive learning evoked by the HFS of the reuniens nucleus are mediated by changes in the short-term plastic changes taking place at the presynaptic terminals of reuniens projecting neurons during the learning process. Additional in vitro experiments are needed to confirm this hypothesis.

#### Proposed roles of the reuniens nucleus in learning processes

It has been postulated that the thalamic midline nuclei comprises a pivotal structure which relays the information from the mPFC to the hippocampal (and parahippocampal) regions [18]–[20]. Of the numerous nuclei of the midline thalamus, the reuniens nucleus is the least known. However, it constitutes the main source of thalamic input to the hippocampus and to the entorhinal cortex, and is strongly interconnected with the prefrontal cortex [17], [30]. More recently, it has been proposed that the reuniens nucleus acts as a relay nucleus of mPFC projections into hippocampal structures [20]. In this regard, the reuniens, and other midline thalamic nuclei, contribute to the proper communication between two functionally interconnected areas – namely, the prefrontal cortex and the hippocampal and parahippocampal circuits, both of them involved in learning and memory processing as well as in other emotional and goal-directed behaviors [19], [20]. Indeed, it has been reported that neurotoxic lesions of the reuniens nucleus affect non-mnemonic aspects of spatial orientation in the water maze test [31]. In contrast, it has also been reported that both reference and working memories in the water maze test are affected by the reversible inactivation of the reuniens nucleus of rats [32]. According to the present results, HFS of the reuniens nucleus delays the proper and complete acquisition of both associative learning (classical conditioning of eyelid responses) and of a cognitive test (object recognition). These results support the proposal that reuniens nucleus gates information flow between the hippocampus and the mPFC [20].

In any case, the reuniens nucleus seems to play a pivotal role in different aspects of memory and learning processes involving the mPFC and the hippocampus [33]. A putative mechanism underlying reuniens nucleus effects on learning and cognition is discussed below.

### A putative presynaptic modulation of the reuniens-mPFC and reuniens-CA1 synapses is involved in reuniens nucleus role in associative learning

In an early study [34] carried out on hippocampal slices it was reported that HFS is able to modify paired-pulse facilitation, indicating a putative involvement of presynaptic mechanisms in the generation and maintenance of early (<1 h) and late (>1 h) LTP. However, this is a proposal that has been either confirmed [29], [35], [36] or refuted [37]–[39] in many other studies. It is also important to bear in mind that paired-pulse facilitation can also be modified by post-synaptic changes evoked by HFS [40]. It must be pointed out that in most of those studies, alterations in paired-pulse ratio were monitored up to a few hours after HFS, and no information could be provided by later changes. In contrast, the evolution of paired-pulse modulation was followed here up to three days after HFS of the reuniens nucleus. In this way, we have provided a definite picture of changes in short-term potentiation evoked by HFS sessions. According to our results, HFS evoked an initial decrease in paired-pulse facilitation that was slowly recovered in the following days.

According to the residual calcium hypothesis [41], calcium entry during the first spike causes facilitation whether or not transmitter is released [26]. Other presynaptic mechanisms interfering with vesicle release are changes in action potential duration, modulation of presynaptic calcium channels, inactivation of calcium currents, etc. [28], [42]. In contrast, paired-pulse depression seems to be due to a reduction in the number of available transmitter quanta at presynaptic sites [26], [43]. Moreover, different types of presynaptic receptor produce specific excitatory or inhibitory effects on transmitter release [28], [42], [44], [45]–[48]. The elaborate organization of interneuronal circuits acting presynaptically on the reuniens-mPFC and reuniens-CA1 synapses would certainly help to explain this form of short-term homeostatic modulation of synaptic strength, making possible its peculiar contribution to the acquisition of this type of associative learning task [49]–[51]. It is then possible to suggest that HFS of the reuniens nucleus interferes with the proper acquisition of a classical eyeblink conditioning task by disrupting ongoing short-term synaptic changes taking place in reuniens nucleus projections to selected cortical sites, including the mPFC and the hippocampal pyramidal CA1 area.

It has been shown recently that the experimental induction of LTP at different stages of conditioning disturbs the acquisition (or extinction) process, when evoked at the hippocampal CA3-CA1 synapse [2], [5]. Moreover, changes in synaptic strength evoked by the learning process are able to interfere with LTP induced subsequently by HFS of the involved hippocampal neural circuits [52]. The present results offer a new conceptual approach to the understanding of neural synaptic processes underlying learning and memory. As shown here, the short-term modulation of synaptic processes following HFS or recorded during associative learning could be used as an index of use-dependent synaptic changes taking place at selected hippocampal [25] and thalamo-cortical (this paper) synapses.

Nevertheless, there is an alternative explanation for the effects of HFS of the reuniens nucleus on the acquisition of associative and cognitive learning. It has been proposed that reuniens axons projecting to the CA1 area terminate on GABAergic interneurons establishing asymmetric (i.e., excitatory) synapses [17], [30], . The diminution of short-term potentiation evoked by the HFS of the reuniens nucleus could modify the delicate inhibitory control of hippocampal inhibitory interneurons on pyramidal CA1 neurons, for example, by evoking an occlusion phenomenon, through an excessive firing of pyramidal cells. In short, the inappropriate firing of pyramidal CA1 neurons would prevent the proper acquisition of associative learning tasks [2].

## Materials and Methods

### Experimental subjects

Experiments were performed on adult male Swiss mice (3- to 5-month-old; 28–35 g) obtained from an authorized supplier (Animal House of the University of Granada, Granada, Spain). Mice were kept on a 12-h light/dark cycle with constant ambient temperature (21±1°C) and humidity (50±7%). Food and water were available *ad libitum*. Electrophysiological and behavioral studies were performed in accordance with the guidelines of the European Union Council (2003/65/CE) and Spanish regulations (BOE 252/34367-91, 2005) for the use of laboratory animals in chronic experiments. Experiments were also approved by the institutional committee for animal care and handling (Vicerrectorate of Research/Ethic Committee Code 13/01/11).

The experimental animals were assigned to the following groups: (i) animals having stimulation in the reuniens nucleus and fEPSP recordings in the prefrontal cortex (n = 20); these animals were classically conditioned with a trace paradigm in the presence (n = 10) or absence (n = 10) of two HFS sessions; (ii) animals stimulated in the reuniens nucleus and with fEPSP recordings carried in the hippocampal CA1 layer (n = 20); half of these animals were presented with two HFS sessions before the first two conditioning sessions; (iii) mice (n = 20) presented with an object discrimination test in the presence (n = 10) or absence (n = 10) of an HFS stimulation session; (iv) animals (n = 10) presented with two HFS sessions, and the fEPSPs evoked by pairs of pulses (40 ms of interval) in the PFC and in the CA1 were followed for up to 3 d; and (v) ten additional animals used for the histological study.

### Surgery for chronic experiments

Once anesthetized (ketamine, 35 mg/kg and xylazine, 2 mg/kg, i.p.), mice were placed in a multi-arm stereotaxic frame. Body temperature was maintained at 37°C with a water blanket. A pair of stimulating electrodes was implanted stereotactically in the right reuniens nucleus (−0.82 mm posterior, +0.2 mm lateral, and −4.5 mm below bregma [54]). These bipolar stimulating electrodes were made from 50 µm, tungsten Teflon-coated wire (A-M Systems, Carlsborg, WA). Separation between tips was <1.0 mm, with the cathodic tip ∼0.5 mm ventral to the anodic one. For experiments included in groups (i) and (ii), animals were also implanted with a recording electrode aimed at the right mPFC (+1.94 mm anterior, +0.25 mm lateral, and −3.12 mm below bregma) for half of the animals or at the right CA1 layer of the hippocampus (−3.16 mm posterior, +3.2 mm lateral, and −2 mm below bregma) for the other half. The mPFC and CA1 recording electrodes were made from a single Teflon-coated tungsten wire (A–M Systems). The final depths of stimulating and recording electrodes were adjusted to achieve a maximal fEPSP response [2], [5].

In the same surgical step, animals included in groups (i) and (ii) were implanted with four electrodes in the upper eyelid of the left eye. Electrodes were also made from 50 µm, Teflon-coated, annealed stainless steel wire (A–M Systems), with their tips bared of the isolating cover for 0.5 mm and bent as a hook. Two of the electrodes were aimed at the supraorbital nerve, and served for the application of electrical stimuli. The second electrode pair was implanted in the ipsilateral orbicularis oculi muscle and served for recording its EMG activity. Another electrode was soldered to a screw and fixed in the skull, serving as ground.

The eight electrodes were soldered to two 4-pin sockets (RS-Amidata, Madrid, Spain) and the whole assembly was fixed with dental cement to the cranial bone. After surgery, animals were kept in independent cages, with free access to food and water, for the rest of the experiment. Experiments were started one week after surgery.

### Recording and stimulation procedures

The EMG activity of the orbicularis oculi muscle was recorded with Grass P511 differential amplifiers (Grass-Telefactor, West Warwick, RI, USA) at a bandwidth of 0.1 Hz–10 kHz. Field EPSP recordings were also made with Grass P511 differential amplifiers through a high-impedance probe (2×10^12^ Ω, 10 pF). For input/output curves, animals were stimulated in the reuniens nucleus with paired pulses (40 ms of interstimulus interval) at increasing intensities (0.1–1.8 mA). The effects of paired pulses at different (20, 40, 100, 200, and 500 ms) interstimulus intervals were also checked. In this case, we used intensities corresponding to 35-50% of the amount necessary to evoke a saturating response [25]. To avoid any cumulative effect, intensities and intervals were presented at random. For the range of intensities used here, population spikes were observed rarely in the collected recordings.

For LTP induction, each animal was presented with one or two HFS sessions. An HFS session consisted of five 200 Hz, 100 ms trains of pulses at a rate of 1/s. This protocol was presented six times, at intervals of 1 min. Thus, a total of 600 pulses were presented during the HFS session. Unless otherwise indicated, and in order to avoid evoking large population spikes and/or the appearance of EEG seizures, the stimulus intensity during HFS was set at the amount necessary to evoke about 1/3 of the maximum fEPSP response (0.4–0.9 mA) – that is, well below the threshold for evoking a population spike [2], [55]. An additional criterion for selecting stimulus intensity was that a second stimulus, presented 40 ms after a conditioning pulse, evoked a larger (>20%) synaptic field potential than the first [2], [56]. In addition, fEPSP evolution after HFS sessions was checked with pairs of pulses (40 ms of interval) presented to the reuniens nucleus for up to 72 h following the first HFS session.

### Classical conditioning procedures

Conditioning sessions were carried out with two animals at a time. Animals were placed individually in a small (5×5×10 cm) plastic chamber located inside a larger (30×30×20 cm) Faraday box to eliminate electrical interferences. A trace conditioning paradigm was used. For this, animals were presented with a tone (2400 Hz, 70 dB, 20 ms) as CS, followed 500 ms later by an electrical stimulation (250 µs, 3 x Threshold, cathodic pulse) as US. Pairs of CS-US presentations were separated at random by 30±5 s. In total, two habituation, 10 conditioning, and three extinction sessions were carried out per animal. A conditioning session consisted of 60 CS–US presentations, and lasted ∼30 min. In order to determine the complete EMG profile of CRs, in 10% of the cases the CS was presented alone. For habituation and extinction sessions, only the CS was presented, also for 60 times per session at intervals of 30±5 s. As a criterion, we considered a “CR” the presence, during the CS-US interval, of EMG activity lasting ∼10 ms and initiated ∼50 ms after CS onset. In addition, the integrated EMG activity recorded during the CS-US interval had to be at least 2.5 times greater than the averaged activity recorded immediately before CS presentation [2], [21].

Synaptic field potentials in the mPFC or in the hippocampal CA1 layer were evoked during habituation, conditioning, and extinction sessions by a single 100 ìs, square, biphasic (negative–positive) pulse applied to the reuniens nucleus 250 ms after CS presentation. Stimulus intensities ranged from 0.4 mA to 0.9 mA.

### Object recognition task

In the object recognition task, mice were individually habituated to an open field (40×25×15 cm), under low-illumination conditions and with no objects, for 5 min. Mice behavior was videotaped using a digital camera (Airis N729, Madrid, Spain) mounted 1 m above the open field. During the training session, two unknown but identical objects (O1 and O2) were put into the open field, and the mouse was allowed to explore them freely for 10 min. The time spent exploring each object and the total approach time (i.e., the time spent exploring both objects) were quantified. As criterion, object exploration was determined as the time spent by the animal close to the object and touching it with the nose or the vibrissae. After each trial, the apparatus and the objects were thoroughly cleaned with 70% ethanol. One hour after the first training, mice were allowed to explore the open field for another 10 min, when one of the two familiar objects (O1 or O2) was replaced with an identical object (O3), and the other (O1 or O2) with a novel object (B1). The time spent exploring each object and the total approach time were quantified again. Within each experimental group, the object positions were interchanged between mice to avoid location bias. After 24 h, mice were tested again, with a new object (C1) and an object identical to the previous one (B2).

### Histological study

At the end of the behavioral studies, mice were divided in three groups: naïve control animal (without electrode), electrode controls (animals without stimulation) and stimulated animals (animals with stimulation). Mice were deeply re-anesthetized (sodium pentobarbital, 50 mg/kg), and perfused transcardially with saline and 4% phosphate-buffered paraformaldehyde. Selected sections (50 µm) including the thalamus, ventral hippocampus, and prefrontal cortex were mounted on gelatinized glass slides and stained using the Nissl technique with 0.1% cresyl violet. That allowed us to determine first, the exact location of the stimulating and recording electrodes in the brain (Fig. 1) and second, to verify whether neuronal loss or atrophy occurs in the reuniens nucleus. Quantification of neuronal density and reuniens nucleus area was carried out using the free Image J Software.

### Data collection and analysis

For the sake of homogeneity, only data collected from animals that completed all the required tests were stored and analyzed (n≥7 per group and task). Data were stored directly on a computer through an analog/digital converter (CED 1401 Plus, Cambridge, England), at a sampling frequency of 11–22 kHz and an amplitude resolution of 12 bits. Data were analyzed off-line for quantification of CRs, fEPSP amplitude, and object recognition times with the help of homemade representation programs [2], [21], [23].

Collected eyelid data were quantified, through a purpose-designed Excel worksheet, as the percentage of CRs per session — namely, the proportion of stimulations within a session of 60 presentations that generated an EMG activity satisfying the above-mentioned criteria. For fEPSP analysis, five successive evoked field synaptic potentials were averaged, and the mean value of fEPSP amplitude was determined between the lower and upper inflection points of the evoked field potential [2]. Time expended on each object during the object recognition test was quantified from the collected videos.

Statistical differences between groups were compared across conditioning and extinction sessions using the two-way repeated measures analysis of variance (ANOVA) test, performed with the SPSS 13.0 for Windows package (SPSS Inc, Chicago, IL). Unless otherwise indicated, data are represented by the mean ± s.e.m. Collected data were analyzed using a two-way ANOVA test, with time or session as repeated measure, coupled with contrast analysis when appropriate. One-way ANOVA allowed checking the statistical differences between different groups. In all of the cases, the corresponding statistical significance test (i.e., F_[(*m*-1), (*m*-1) x (*n*-1)]_ statistic) were reported where *m* and *n* indicate number of groups and number of animals, respectively.
